# The Effect of Spacer Treatment of Infected Hip and Knee Arthroplasties on Patients’ Mental Health: A Narrative Review of the Literature

**DOI:** 10.3390/healthcare12070790

**Published:** 2024-04-06

**Authors:** Donato Di Gennaro, Giannantonio Coletta, Enrico Festa, Domenico De Mauro, Maria Rizzo, Luca Diana, Giovanni Balato, Massimo Mariconda

**Affiliations:** 1 Orthopedic Unit, Department of Public Health, “Federico II” University, 80131 Naples, Italy; donato.digennaro@unina.it (D.D.G.); giannantonio.coletta@unina.it (G.C.); enrico.festa@unina.it (E.F.); maria.rizzo@unina.it (M.R.); luca.diana@unina.it (L.D.); maricond@unina.it (M.M.); 2 Department of Orthopedics and Geriatric Sciences, Fondazione Policlinico Universitario A. Gemelli IRCCS, 00168 Rome, Italy; demaurodomenico@gmail.com

**Keywords:** mental, spacer, periprosthetic joint infections, PJI, infection, health, proms

## Abstract

Background: The gold standard treatment for periprosthetic joint infections is the two-stage revision that includes the spacer placement before definitive reimplantation. The management of PJI affects patients’ joint function and, subsequently, their mental health. Even though significant advances have been achieved, little to no attention has been paid to the psychological implications. So, based on standardized patient-reported outcome measures (PROMs), this study aimed to clarify the effect of spacer treatment of infected hip and knee arthroplasties on patients’ mental health. Methods: We performed research on the literature on PJIs in the English language using the MEDLINE database with the search strings “spacer” OR “spacers” AND “hip” OR “knee” AND “SF-12” OR “SF-36” OR “EQ-5” OR “mental” OR “depression” OR “anxiety.” The reference lists of selected articles were also hand-searched for any additional articles. Results: A total of 973 published papers were extracted, and 9 papers were finally included. A total of 384 patients who underwent spacer placement for PJI were identified. Of these 384 patients, 54% were female. The mean age ranged from 62 to 78.2 years. Of the11 papers identified for this review, 4 analyzed only hip spacers, including 119 patients; 4 only knee spacers, evaluating 153 patients; while a single study included 112 patients for both joints. Conclusions: Patients with the spacer are living in a state of mental upset, albeit better than the preoperative state. Clinical improvement with the review is not assured. The alteration of mental state turns out not to be transient for all the patients.

## 1. Introduction

Worldwide periprosthetic infections represent one of the most fearsome complications after total knee and total hip arthroplasty, with their importance indicated by the fact that besides being common causes of revision, the first for the knee (16.8% of all knee revisions) and the third for the hip (14.8% of all hip revisions), they also pose a significant challenge to patients, orthopedic surgeons, and the healthcare system.

This kind of infection not only impairs joint function but also engenders systemic repercussions, leading to prolonged hospitalization, repeated surgeries, increased healthcare costs, and the potential for long-term disability [1]. Furthermore, it can seriously impact patients who need more physical, psychological, and social support [2]. 

Although the evaluation of the influence on psychological status is often underestimated, the management of PJI affects patients’ mental health and overall well-being because the path from diagnosis to treatment and recovery is fraught with uncertainty, engendering feelings of helplessness and distress [3]. Treatment could involve additional surgeries, mechanical and chemical debridement of the bacterial biofilm that infects bone and soft tissues and contaminates the prosthetic implant, intravenous antibiotics, and eventually, rescue procedures, loss, or at least reduction of social participation [4,5,6,7]. After the explantation of the infected arthroplasty, most patients experience a restriction of joint mobility with the use of a cast or brace, and some patients cannot walk. This is coupled with a long stay with reduced personal contact and isolation. Functional limitations for the operated limb, the alteration of the daily activities for the long stay, and the uncertainty of the prognosis can contribute to a change in the mental health of these patients. Generally, anxiety and depressive spectrum disorders, as well as frustration, are the most critical emotional challenges that individuals grappling with PJI may encounter [8,9,10]. The psychological aspect and its consequences have lagged behind, not evaluated and deepened, regarding the remarkable steps ahead that have been made in order to improve the functional and infectious outcome of these patients. So, based on standardized patient-reported outcome measures (PROMs), this study aimed to clarify the effect of spacer treatment of infected hip and knee arthroplasties on patients’ mental health.

## 2. Materials and Methods

To identify relevant papers dealing with the mental health of patients after spacer implantation for hip or knee arthroplasty infection, we performed research on the literature on PJIs in the English language using MEDLINE, like Embase, PsycINFO, and Cochrane Library as databases, with the search strings “spacer” OR “spacers” AND “hip” OR “knee” AND “SF-12” OR “SF-36” OR “EQ-5” OR “mental” OR “depression” OR “anxiety” OR “HADS” OR “PHQ-9” OR “HAQ”. The reference lists of selected articles were also hand-searched for any additional articles that were not identified from the database search. The inclusion criteria were not limited to English language literature and specific publication dates.

### 2.1. Eligibility Criteria

Longitudinal studies (retrospective and prospective) and randomized controlled trials evaluating the mental health of patients after spacer implantation were finally selected. Papers that considered hip and knee periprosthetic infections were also included. The exclusion criteria included case reports, expert opinions, previous metanalysis and systematic reviews, letters to the editor, and studies that did not report patient-reported outcome measures (PROMs).

### 2.2. Study Assessment and Data Extraction

Initially, the titles and abstracts of the studies were screened by two independent reviewers (DDG and GC). The full text was obtained for articles whose abstracts met the inclusion criteria or those without any uncertainty. Then, each study was assessed based on the inclusion criteria by two independent reviewers (EF and DDM), and any disagreement regarding inclusion of a particular study was resolved by evaluation of the article by the senior authors (LD, MR, GB and MM). Relevant data were extracted from each study. Data on participant demographics, sample size, type of spacer, failure outcomes, and clinical and functional outcomes were recorded.

## 3. Results

**Figure 1:**
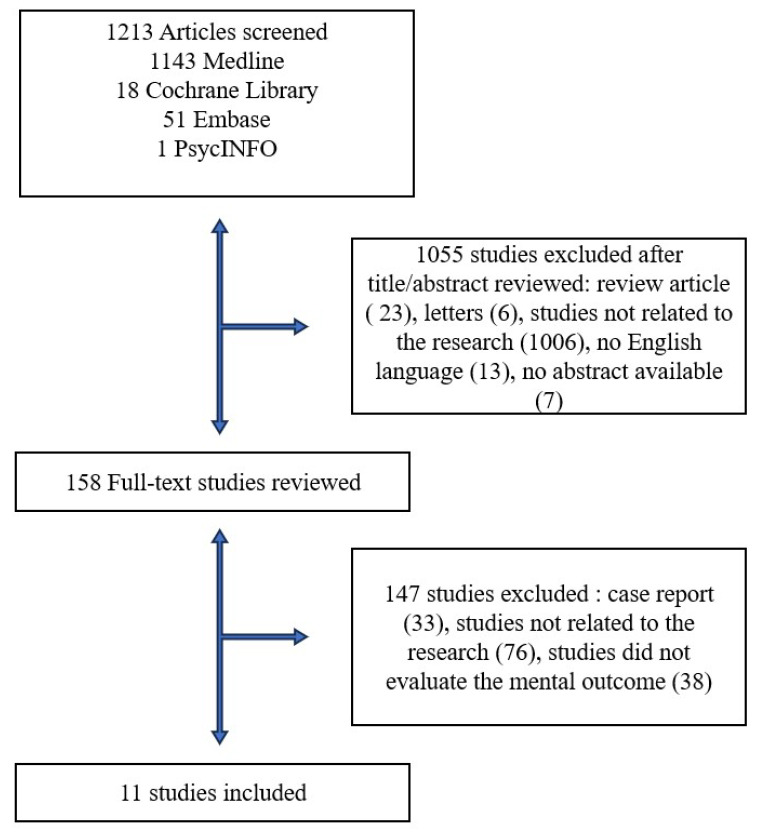
Flow chart of studies identified for the review.

A total of 1213 published papers were extracted. Overall, 1202 papers were excluded from the analysis for the following reasons: 23 were review articles, 13 were not in English, 33 were case reports, 1082 studies were not related to the research, 38 studies did not evaluate the mental outcome, 6 were letters to the editor, and 7 papers had a missing abstract. Eleven papers were finally included (Figure 1). A total of 384 patients who underwent spacer placement for PJI were identified. Of 469 patients, 56% were female [3,11,12,13,14,15,16,17,18,19,20]. The mean age ranged from 62 to 78.2 years. Of the eleven papers identified for this review, four [11,12,13,14] analyzed only hip spacers, including 119 patients; six [3,16,17,18,19,20] only knee spacers, evaluating 238 patients; while a single [15] study included 112 patients affected by hip and knee periprosthetic joint infections. The demographic data of the selected studies are shown in Table 1. 

### 3.1. Hip

All the analyzed papers [11,12,13,14] have evaluated the use of articulated dynamic spacers. Among them, Lunz et al. have studied custom-made spacers, Beaupre et al. and Scharfenberger et al. spacers with the Prostalac system instead, while Rollo et al. have evaluated both preformed spacers and custom-made spacers. In this case [11], the spacer was constructed using carefully molded antibiotate cement to recreate an acetabular cavity as functional as possible in terms of the orientation and size, associated with a cemented stem with a metal head. Beaupre et al. and Scharfenberger et al., have instead evaluated the Prostalac system in which, through special molds, it is possible to implant cemented femoral components, carefully sized, and acetabular components, all-poly, also cemented in this other case [12]. The spacers evaluated were as follows: hand-made with concrete modeled to recreate the femur and the head, reinforced with Steinmann pins to avoid breakage, and preformed already available in various sizes (Vancogenx-Space Hip). Except for Beaupre et al., all the studies reported mechanical complications occurring in a percentage varying between 11% [14] and 31% [12]. The average retention time of the spacer was 226 days, with a range varying between 90 [11] and 360 [14] days. Lunz et al. and Rollo et al. have shown high conversion rates to the second stage, 96% and 100%, respectively; Beaupre et al. reported a conversion rate of 32%. According to the state of mental health assessment, four different questionnaires were used: Veterans RAND 12-Item Health Survey (VR-12); Short-Form 12-item Health Survey (SF-12); RAND 36-Item Health Survey (RAND-36); Short-Form 36-item Health Survey (SF-36). The VR-12 is a brief, self-administered health survey comprised of 12 items. The 12 items in the questionnaire correspond to 8 principal physical and mental health domains and are summarized into 2 scores, a Physical Component Scale (PCS) and a Mental Health Component Scale (MCS). The SF-12 is also a self-administered health survey with 12 items. The score is almost identical to the VR-12, with the latter being a derivative of the SF-12, from which it differs only in some answers and the resulting scores. The SF-36 and the RAND-36 turn out to be the extended original versions, with 36 items, of the SF-12 and VR-12, respectively. In all these cases, the items are summarized in the two scale, PCS and MCS. Three studies [11,12,13] have shown a clinical improvement compared to the preoperative status; this improvement was shown to be progressive with the increase of the follow-up in the cohort of Rollo et al., reaching the value of 86.1 and 86.9 points for the preformed and custom-made spacers at the last reported control of 12 months, respectively, with the SF-12 questionnaire. Lunz et al. observed a statistically significant improvement with the spacer implantation. Still, the revision does not seem to confirm this improvement, reporting a score of 43 points for the mental health component scale (MCS), with the VR-12 questionnaire, at the last follow-up, the same as the three-month follow-up with the spacer in situ. Beaupre et al., on the contrary, observed that clinical improvement was not obtained with the revision, reporting better data for patients who had retained the spacer than those who made the second stage at 24 months, even if these differences were not statistically significant, based on data for the MCS of RAND-36 questionnaire. Scharfenberger et al. compared spacer patients with two different cohorts, patients with arthrosis of the hip and patients six months post primary total hip replacement (THR). The authors observed how the values for mental health status, with the MCS of SF-36 questionnaire, of the patients with a Prostalac spacer were no different from the arthrosis population but statistically significantly lower than the primary THR group, 66.13 and 76.1 (p 0.03), respectively, highlighting the positive effect of the spacer in the return to a pre-infectious state, from the mental point of view, although not at the levels of an uncomplicated prosthesis. The characteristics of the studies analyzed for hip joints are summarized in Table 2.

### 3.2. Hip and Knee

Furdock et al. compared a group of septic revisions, including static and articulated hip and knee spacers, with a group of aseptic revisions [15]. The authors did not provide further information on the manufacturing of these spacers, whether they were preformed or custom-made. However, in the group of so-called static was applied a ban on loading throughout the interim period. They reported no mechanical complications, with a conversion rate to the second stage of 77% after an average retention period of 120 days. Their analysis, conducted through the Patient-Reported Outcomes Measurement Information System Depression Score (PROMIS), showed that patients in the two-stage group had a statistically significantly higher rate of onset of a major depressive disorder (MDD) than the control group, 20%, and 6.4%, respectively. PROMIS is a T-score metric. High scores mean a high value of the measured variable. Ten points on the T-score metric is one standard deviation (SD). A score of 40 is one SD lower than the mean of the reference population; instead, a score of 60 is one SD higher than the mean of the reference population. At the same time, the preoperative scores were worse in the septic group than in the aseptic one. These scores gradually improved until reaching the levels of the aseptic group at the last follow-up, without any statistically significant differences. Regarding spacers, the interim period scores were comparable with the preoperative scores, with no statistically significant differences between the static and articulated groups. The authors reported, in addition, that the lower consumption of preoperative antidepressants and the time since the final intervention are factors that correlate with lower depression scores. The characteristics of the studies analyzed for hip and knee joints are summarized in Table 3.

### 3.3. Knee

Regarding the knee spacers, three of the six papers have evaluated different types of spacers [16,18,19]; the other three have instead studied a single kind of spacer [3,17,20]. In particular, Helito et al. analyzed articulated spacers and non-articulated spacers. For non-articulating, we do not mean static because, in this case, a certain degree of movement was allowed. More particularly, the non-articulating spacer was created by affixing antibiotate cement between the articular surfaces of the femur and tibia, in the absence of specific components of cement, metal or reinforcement elements; on the contrary, for the articulated spacer, the authors did not provide information on the manufacture of these spacers but reported an extremely small range of movement between 5° and 45° degrees. Preobrazhensky et al. compared states and articulation. In this case, the authors have used, for articulated spacers, the same clean and re-sterilized explanted components associated with antibiotate cement suitably positioned at a more advanced stage of polymerization to better manage and fill gaps; the static spacers were hand-made with the same cement applied with reinforcing rods and inserted in the epiphysis distal femur and tibia. Similarly, Lee et al. compared states and articulations; however, the authors have not reported any information about the spacers’ constitution. Instead, Knebel et al. and Zamora et al. evaluated only articulated and Walter et al. only static spacers. In one case [3], the authors described their spacers as multi-part articulating spacers, thus providing no additional information about the nature of the components or the surgical technique. In the other case [20], the authors composed their spacers of a PS femoral component (NexGen; Zimmer Biomet, Warsaw, IN, USA) and an all-polyethylene PS tibial component or a standard PS tibial insert without a metal base-plate The static ones instead [17] were made with two humeral nails (T2, Stryker, Duisburg, Germany), which are available in small diameters and are well suited to be covered with antibiotate cement. The nails were wrapped with cerclage wire to avoid the debonding of the cement. Subsequently, after the cement was applied and completed polymerization, the two nails were inserted into the distal epiphyses of the femur and tibia, maintaining a certain overlap at the joint level. The central defect was then filled with other antibiotate cement. Of the studies analyzed, only one case reported the rate of mechanical complications, which was attested to around 7% [17]. As for the retention time of the spacer, this varied between 45 d [3] and 210 d [18], with an average of 126 d. Two of the six papers reported [3,18] revision to the second stage, with the rates ranging between 100% and 88%, respectively. The mental status was evaluated with eight questionnaires: Patient Health Questionnaire-4 (PHQ-4); Short-Form 12-item Health Survey (SF-12); Questions on Life Satisfaction-Health Life Satisfaction (FLZ-HLS); Fear of Progression Questionnaire (PA-F-KF); Short-Form 36-item Health Survey (SF-36); EuroQol Group-5D (EQ-5D); ICD-10-based symptom rating (ISR); Hospital Anxiety and Depression Scale (HADS). The PHQ-4 is a four-item patient self-assessed questionnaire for anxiety and depression. Scores are rated as normal (0–2), mild (3–5), moderate (6–8), and severe (9–12). A total score ≥ 3 for the first two questions suggests anxiety. A total score ≥ 3 for the last two questions suggests depression. The FLZ-HLS is a self-assessed questionnaire comprising 16 items divided into two modules, “General Life Satisfaction” and “Satisfaction with Health”. The respondent rates each items twice, once for the subjective importance of the aspects of life or health addressed, and once for the degree of satisfaction in that area. The two ratings are combined into a total score. The PA-F-KF is the short form of the Progressive Anxiety Questionnaire, a self-assessment tool used to screen for stress in patients with progressive anxiety. This questionnaire consists of 12 items. For evaluation, the scores are comprised in a range of 12–60, with values higher than 34 regarded as critical. The EQ-5D is a self-assessed questionnaire used to evaluate the quality of life. The system is subdivided into five dimensions, with one question for each of these. The answers can be converted into an EQ-5D score that ranges from 0, for death, to 1, for perfect health. The ISR was developed to measure status and change on the basis of self-assessments. The basis of the instrument are the symptoms listed in Chapter F of the ICD-10, It comprises 29 questions in 6 subscales, including depressive disorders syndrome and anxiety disorders syndrome. The HADS has a maximal score of 42 that is composed of scores from 0 to 21 each for anxiety and depression. The interpretation of the HADS scores is as follows: normal (0–7), borderline normal (8–10), abnormal (11–21). The data reported by Knebel et al., relating to the PHQ-4, showed a fluctuating trend in the anxiety of patients with an intermediate preoperative state, an improvement after spacer implantation, and a subsequent worsening before revision. The analysis of the SF-12 seems to suggest a progressive deterioration in the mental component equally. In both cases, the values are higher at the last follow-up than those of the preoperative state. The authors reported only the mean value for the other two questionnaires used, the FLZ-HLS and PA-F-KF. The FLZ-HLS showed a worse mental condition than the reference population of the study, the general German population according to the authors, with significantly lower values (*p* = 0.075). Regarding the PA-F-KF, with an average score of 31.24 points, patients’ most significant concern was being dependent on outside help. In Helito et al.’s analysis, spacer retention was compared to amputation as a rescue procedure. After 35 months of follow-up, the authors found a score of 47.1 points, with the SF-36, a value worse than amputees’, 49.9 points. An opposite evaluation was performed instead for the functionality. Two studies evaluated the EQ-5D; in particular, Walter et al.’s data seem to be compatible with Preobrazhensky et al.’s data, with 0.36 found for static spacers at three days of follow-up, and intermediate data between 0.21 preoperative and 0.56, reported by Preobrazhensky et al., at six months of follow-up, for the static group. These data help to suggest a progressive improvement of the mental state in this subgroup of patients. According to the authors [18], this improvement would always be parallel to the articulated ones, which had better scores during the interim period, reaching values identical at the last follow-up. Walter et al. reported a statistically significantly higher rate of depression disorder with the ISR when compared with a control population, represented by a group of patients previously successfully treated. Lee et al. reported a significant improvement in both anxiety and depression in both groups, articulate and static, four weeks post-surgery compared to the preoperative scores. However, there were no differences between the static and articulated scores. As for the SF-36, there was an improvement in the score only in the articulated group, but compared to other studies that used the same score [16,17], the score was lower, even considering the significant differences in timepoints. Zamora et al. compared the results of the retained articulated spacers and the two-stage revisions, showing no statistically significant difference, 49.7 and 47.1, respectively, at three years of follow-up. This score, with the SF-12, turns out to be the same as that achieved at three months post-revision by the group of Knebel et al. The characteristics of the studies analyzed for knee joints are summarized in Table 4.

## 4. Discussion

Depression, anxiety, and other mental state alterations are common in patients undergoing primary and revision arthroplasty, with a prevalence varying between 15% and 20% [21,22]. In particular, in the context of septic revisions, the prevalence is higher, exposing the patient to higher rates of complications [23,24]. From this point of view, the main finding of our paper is the observation that patients with the spacer live in a state of mental health upset, albeit better than the preoperative state. In more detail, regarding the normative data of the populations examined in the analyzed papers [25,26,27,28,29,30,31,32], of the seven works that provided data on the pre-operative state, all reported a worse mental condition than the normal population [3,11,12,13,15,18]. This condition is well known in the literature; several authors have, in fact, highlighted the higher rate of anxiety and depression in patients diagnosed with PJI, also analyzing how the same mental state is related to the greater risk of infection [15,17,33,34,35,36,37]. From this point of view, our review suggests that although the mental state appears to be compromised in all the analyzed works, these conditions could be transitory with a certain improvement over time. The extent of this improvement is reported to be extremely variable. As regards the hip, Rollo et al. reported a progression with a difference of about 40 points with the SF-12 at one year of follow-up without significant difference between different types of spacers, 86.1 and 86.9, respectively, for the preformed group and the homemade group, while the score was near to 90.4 for the Italian normal population used as reference [26].

However, it should be considered that the authors reported only the total value of the SF-12 questionnaire, meaning that the mental component score could be overestimated. Lunz et al. described an improvement of about 5 points with the VR-12, reaching 43 points, 9 points lower than the reference German population, but without a progression with the revision, sustaining an unchanged score at one year of follow-up. Instead, Beaupre et al. observed a progressive improvement that continued with the revision and even higher for the patients not reviewed. These values are higher than those of the normal population, the Canadian population. However, it should be considered that in this case, the reference parameters used are those of the SF-12 and SF-36, missing the normative values of the VR-12 and RAND 36 for German and Canadian populations [26,27,28,30].

Scharfenberger et al. have observed that there is still a statistically significant difference, compared to the control group with primary prostheses, at one year of follow-up; this helps to suggest a transient deficit of mental status but not of a short duration. At the same time, they observed no differences from the arthritis control population group, highlighting the positive effect of the spacer in returning to a mental state compatible with the pre-infectious state, even if not at the level of the uncomplicated prostheses.

The study of Furdock et al. was the only one that included both knees and hips; however, the authors did not report a different score for the two joints. It was observed that a gradual improvement occurred, albeit with a fluctuating trend. The interim period was the worst, maybe as a consequence of the 82% of static spacers with a ban on loading in this population, which could reflect on the mental state. Also in this paper, the alteration seems to be a transient deficit. At the last follow-up, the authors reported no difference with the control group, patients undergoing aseptic revision procedures, but stressed that septic patients are more prone to the development of depression. Knebel et al. used several questionnaires in their analysis, finding, on the one hand, a progressive but fluctuating improvement with lower data during the interim period with the SF-12 and PHQ-4 for mental state and anxiety, while with the FLZ–HLS and PA-F-KF, the data are substantially unchanged compared to the preoperative, suggesting a less transient non-progressive improvement. Similarly, Preobrazhensky et al. observed, with the EQ-5D, a progressive improvement, with the values at the last follow-up not dissimilar between static and articulating and not far from the normal population, 0.82 to 0.85 for the Russian population [29]. During the interim period, however, the articulated have slightly better values. Lee et al. observed a significant reduction in anxiety and depression, as early as four weeks post-surgery, without a significant difference between static and articulated. They noted also, like Preobrazhensky et al., an improvement for the SF-36, only for the articulated group; however, with values lower than the normative population, indicating a persistent change in mental state [32]. The data reported by Zamora et al. indicate that spacers, in the long term, could have a performance comparable to the revision, having observed no difference when comparing mental health, but also in functional terms, between these two groups. In this case, the group is represented by only articulated spacers. These observations may suggest that at least some patients do not need the second stage. Helito et al., in contrast to these data, observed that the function is not directly related to the mental state, so the control group of amputees not walking with reduced functions had values higher than patients with a spacer, opposed to Furdock et al. However, the characteristics of the spacer, not articulating, especially the very small number of patients, the particular characteristics of the reference population and the very different evaluation times must be considered. Similarly, the data of Walter et al. at three days post-operation, with static spacers, seem to be compatible with those of Preobrazhensky et al. and Lee et al.; however, the great differences in the population and time points must be considered. The authors also reported an increase in the rate of depression in this type of patient, but the short follow-up time could affect these data. It is therefore clear that periprosthetic infections represent a pathology with many facets, some of which, such as the changes in the mental state, do not necessarily resolve with the resolution of the infection. Observations that both in the hip [11,15] and in the knee [3,15]. There may be cases of a fluctuating trend or lack of improvement with the revision, which opens the door to the increasingly widespread 1.5 techniques [38,39], not only from a functional but also from a mental point of view, for the better personalization of the treatment.

According to our knowledge, this is the first review that aims to evaluate the mental condition of patients with the spacer in situ in the more general context of hip and knee periprosthetic infection. Our analysis, however, presents some limitations. In the first place, the great heterogeneity of the studies as regards the populations under examination, the questionnaires used, the types of spacers and the timing of measurement make difficult to compare the results. Even where the questionnaires used are the same, account must be taken of the differences between the application of the questionnaires in such different populations. It goes further to consider the big difference in quality of the individual studies analyzed. It should also be considered that mental health changes such as anxiety and depression are multifactorial conditions. Therefore, another possible limitation of this review is represented by all those perioperative factors not included in this study, such as the type of anesthesia or the presence of blood transfusions, which may help explain more or less transient mental health changes [40,41,42,43,44,45]. Finally, also the different nature of the articulations considered and analyzed here together but each having its own characteristics. It should be noted, however, that the very low availability of data in the literature is considerable. As a further result of this study, the observation that spacers are being placed for a limited period, which is increasingly being called into question, and therefore negligible from the mental point of view, finds in this work a certain opposition, so we hope therefore a greater effort from the scientific community to also deepen this aspect in patients suffering from PJI. We also recommend that given these changes in mental health are only partly attributable to the functional limitation of the affected limb but also affecting other fields than orthopedic, multidisciplinary management would be appropriate. This should include first a screening of the patients at risk, perioperative management to avoid triggers, and then the management of complications with specialists in mental health disease.

## 5. Conclusions

Patients with the spacer are living in a state of mental upset, albeit better than the preoperative state. Furthermore, evidence from this review suggests that clinical improvement of the compromised mental state post-two-stage revision is not guaranteed for all patients. The development of this condition can, therefore, be variable, including non-progressive. It follows that even if the state of living with the spacer can be reduced in time, and therefore made transient, not necessarily transient are the alterations that the PJI diagnosis brings. However, it should be considered that the spacer implantation represents an improvement compared to the pre-operative starting state. Further studies will be needed to clarify the causes of this trend.

## Figures and Tables

**Table 1:** Demographic information of studies identified for the review.

Author	Journal	Joint	No. of Joints/Patients	Age, Mean	Female Gender N (%)
Lunz A, et al. 2022 [11]	Archives of Orthopaedic and Trauma Surgery	Hip	24/24	70	8 (33)
Rollo G, et al. 2020 [12]	Journal of Clinical Orthopaedics and Trauma	Hip	50/50	78.2	35 (70)
Beaupre LA, et al. 2017 [13]	Journal of orthopaedic surgery	Hip	22/22	63.9	9 (41)
Scharfenberger A, et al. 2007 [14]	Canadian Journal of Surgery	Hip	23/23	70.1	13 (57)
Knebel C, et al. 2020 [3]	Surgical Infections	Knee	31/31	69	15 (48)
Furdock RJ, et al. 2022 [15]	Arthroplasty Today	Knee/Hip	112/112	65	45 (40)
Helito CP, et al. 2015 [16]	Prosthetics and Orthotics International	Knee	4/4	71	2 (50)
Walter N, et al. 2022 [17]	Frontiers in Surgery	Knee	14/14	67.7	7 (50)
Preobrazhensky PM, et al. 2019 [18]	International Orthopaedics	Knee	104/104	62	73 (70)
Lee SC, et al. 2016 [19]	Current Orthopaedic Practice	Knee	40/40	69.7	37 (92)
Zamora T, et al. 2020 [20]	The Bone and Joint Journal	Knee	46/45	69	24 (51)

**Table 2:** Clinical information for the hip studies. SD, standard deviation; VR-12, Veterans RAND 12-Item Health Survey; MCS, mental component score; SF-12, Short-Form 12-item Health Survey; RAND-36, RAND 36-Item Health Survey; SF-36, Short-Form 36-item Health Survey; *, measurement range; **, retained spacer; [N/A], not applicable.

Author	Spacer N (%)	Spacer Complications N (%)	Score Used	Preoperative	Postoperative (SD) [Time]	Postoperative (SD) [Time]	Spacer Period Mean	Second-Stage N (%)	Post 2-Stage (SD) [Time]	Difference from Preoperative Score
Lunz A, et al., 2022 [11]	24 (100) Dynamic custom-made	3 (12.5)	VR-12 (MCS)	37 (5.7)	43 (7.3) [3 m]	[N/A]	90 d	23 (96)	43 (4.9) [16 m]	+6
Rollo G, et al., 2020 [12]	26 (52) Dynamic preformed	8 (31)	SF-12	44.6 (28–74) *	52.1 (24–68) * [N/A]	61.8 (40–84) * [6 m]	158 d	50 (100)	86.1 (64–94) * [12 m]	+41.5
	24 (48) Dynamic custom-made	7 (29)		44.8 (28–72) *	51.6 (24–72) * [N/A]	62.8 (40–84) * [6 m]	176 d		86.9 (64–100) * [12 m]	+42.1
Beaupre LA, et al., 2022 [13]	22 (100) Prostalac	[N/A]	RAND-36 (MCS)	51.8 (13.7)	55.5 (10.0) [3–6 m]	57.0 (9.9) ** [24 m]	345 d	7 (32)	54.5 (10.7) [24 m]	+2.7
Scharfenberger A, et al., 2007 [14]	28 (100) Prostalac	3 (11)	SF-36 (MCS)	[N/A]	66.13 (27.2) [12 m]	[N/A]	360 d	[N/A]	[N/A]	[N/A]

**Table 3:** Clinical information for the hip and knee studies. SD, standard deviation; PROMIS, Patient-Reported Outcomes Measurement Information System Depression Score; [N/A], not applicable.

Author	Spacer N (%)	Spacer Complications N (%)	Score Used	Preoperative	Postoperative (SD) [Time]	Spacer Period Mean	Second-Stage N (%)	Post 2-Stage (SD) [Time]	Post 2-Stage (SD) [Time]	Difference from Preoperative Score
Furdock RJ, et al. 2022 [15]	92 (82) Static non-weight bearing 20 (18) Articulating	[N/A]	PROMIS	54.8	55.1 [3 m]	120 d	112 (77)	54.3 [4–5 m]	50.4 [10–22 m]	−4.4

**Table 4:** Clinical information for the knee studies. SD, standard deviation; PHQ-4, Patient Health Questionnaire-4; SF-12, Short-Form 12-item Health Survey; MCS, mental component score; FLZ-HLS, Questions on Life Satisfaction-Health Life Satisfaction; PA-F-KF, Fear of Progression Questionnaire; SF-36, Short-Form 36-item Health Survey; EQ-5D, Euro Qol Group-5D; ISR, ICD-10-based symptom rating; Hospital Anxiety and Depression Scale (HADS); * Values are mean over four time points; ** 95% CI; [N/A] not applicable.

Author	Spacer N (%)	Spacer Complications N (%)	Score Used	Preoperative	Postoperative (SD) [Time]	Postoperative (SD) [Time]	Spacer Period Mean	Second-Stage N (%)	Post 2-Stage (SD) [Time]	Difference from Preoperative Score
Knebel C, et al., 2020 [3]	31 (100) Articulating	[N/A]	PHQ-4	5.74	4.75	6.42	45 d	31 (100)	5.07	−0.67
			SF-12	47.2	46.4	45.3			49.7	+2.5
			(MCS)		[1 d]	[6 w]			14.82 *	[N/A]
			FLZ–HLS						31.24 (9.60) *	[N/A]
			PA-F-KF						[3 m]	
Helito CP, et al., 2015 [16]	3 (75) Non-articulating 1 (25) Articulating	[N/A]	SF-36 (MCS)	[N/A]	47.1 [35 m]	[N/A]	[N/A]	[N/A]	[N/A]	[N/A]
Walter N, et al., 2022 [17]	14 (100) Static	1 (7)	EQ-5D SF-36 (MCS) ISR	[N/A]	0.36 (0.32) 47.1 (18.6) 0.52 (0.20)[3 d]	[N/A]	70 d	[N/A]	[N/A]	[N/A]
Preobrazhensky PM, et al., 2019 [18]	67 (73) Articulating 25 (27) Static	[N/A]	EQ-5D	0.18 (− 0.51–0.64) ** 0.21 (0.16–0.63) **	0.57 (0.16–0.8) ** 0.53 (−0.01–0.74) ** [6 m]	0.71 (0.53–1.0) ** 0.59 (−0.08–0.83) ** [9 m]	180 d 210 d	92(88)	0.82 0.82 [24 m]	+0.64 +0.61
Lee SC, et al., 2016 [19]	20 (50) Articulating 20 (50)Static	[N/A]	HADS-A	13.1 (6.7)	7.4 (4.7)	[N/A]	60 d	[N/A]	[N/A]	−5.7
			HADS-D	12.3 (6.2)	6.9 (4.3)					−5.4
			SF36	18.6 (3.6)	21.4 (3.0)					+2.8
			(MCS)	13.1 (6.7)	8.9 (3.8)					−4.2
				11.6 (4.6)	8.6 (3.8)					−2.7
				18.6 (3.6)	20.2 (5.9)					+1.6
					[4 w]					
Zamora T, et al., 2020 [20]	10 (100) Articulating	[N/A]	SF-12 (MCS)	[N/A]	49.7 (9) [36 m]	[N/A]	[N/A]	[N/A]	[N/A]	[N/A]

## Data Availability

Research data are accessible from corresponding author on request.
